# The Metalloproteinase ADAMTS5 Is Expressed by Interstitial Inflammatory Cells in IgA Nephropathy and Is Proteolytically Active on the Kidney Matrix

**DOI:** 10.4049/jimmunol.2000448

**Published:** 2020-09-11

**Authors:** Scott Taylor, Molly Whitfield, Jonathan Barratt, Athanasios Didangelos

**Affiliations:** Mayer IgA Nephropathy Laboratory, University of Leicester, LE1 7RH Leicester, United Kingdom

## Abstract

In IgA nephropathy (IgAN), IgA immune complexes are deposited in the mesangium and drive inflammation and extracellular matrix (ECM) remodelling. The functional links between IgA deposition, inflammation, and matrix remodelling are not well characterized. We recently performed urine liquid chromatography–tandem mass spectrometry proteomics and identified multiple ECM glycoproteins whose expression and function in IgAN is unclear. None of the urine glycoproteins was regulated in IgAN transcriptomics, indicating that tissue remodelling rather than increased expression might contribute to their presence in urine. To investigate this, we examined the IgAN expression profile of metalloproteinases, enzymes involved in the remodelling of ECM proteins, and noted that the proteoglycanase ADAMTS5 was upregulated in IgAN kidneys. ADAMTS5 accumulated in areas of inflammation, and ADAMTS5^+^ cells were seen in the tubulointerstitium and glomeruli. The enzyme was expressed by CD64^+^ cells and its expression was increased by IL-1 and LPS. Analysis of myeloid cell transcriptomics revealed that ADAMTS5 is enriched in human classical monocytes. ADAMTS5^+^ cells were present in areas of matrix remodelling and associated with ECM proteins lumican, versican, and collagen-4. Liquid chromatography–tandem mass spectrometry proteomics of kidney explants digested with ADAMTS5, identified multiple kidney proteins affected by ADAMTS5 and revealed specific proteolysis of complement C3 and fibronectin associated with IgA on immune complexes. ADAMTS5 processing of immune complex proteins reduced binding to cultured mesangial cells. ADAMTS5 is associated with interstitial inflammatory cells in IgAN and other kidney lesions and fragments relevant extracellular proteins. The proteolytic enzyme might be a new translational target relevant to inflammation and scarring in kidney disease.

## Key Points

- ADAMTS5 is upregulated in human IgA nephropathy lesions.
- ADAMTS5 is related to inflammatory infiltrates in affected kidneys.
- ADAMTS5 digests kidney matrix proteins and cleaves complement C3 and fibronectin.

## Introduction

In IgA nephropathy (IgAN), accumulation of IgA immune complexes in the glomerular mesangium causes localized inflammation, remodelling of the kidney extracellular matrix (ECM), and scarring (1). Although inflammation and concomitant tubulointerstitial and glomerular fibrosis in IgAN are well characterized (2), focused studies on ECM accumulation, proteolysis, and remodelling are scarce. Little is known about how inflammation drives proteolysis and matrix remodelling (3), and few studies have specifically studied the expression and accumulation of classic ECM (4–7) and basement-membrane (8, 9) proteins in IgAN kidneys. Moreover, a small number of studies have examined the expression and possible function of matrix-degrading metalloproteinases (MMPs) in IgAN with a focus on MMP2 and MMP9. MMP1 and MMP7 have also received limited attention (10, 11). Nothing is known about a disintegrin and metalloproteinase with thrombospondin motifs (ADAMTSs) in IgAN, the other important group of MMPs involved in glycoprotein and proteoglycan processing in different diseases (12). In inflammation, MMPs and ADAMTSs drive matrix proteolysis and play a significant role in tissue remodelling.

While studying the expression of matrix glycoproteins that we recently identified in IgAN urine by proteomics (13), we noticed the upregulation of ADAMTS5, a matrix enzyme typically associated with proteoglycan digestion in cartilage and other tissues (14, 15). To our knowledge, the presence of ADAMTS5 in IgAN kidneys is described in this study for the first time, together with tissue expression and likely proteolytic effects on the kidney proteome.

## Materials and Methods

### Fluorescence immunohistology

For tissue staining, we used archived kidney biopsies with a proven diagnosis of primary IgAN from 20 patients (12 males and eight females; mean age 51 ± 16) admitted at the John Walls Renal Unit, Leicester General Hospital. IgAN biopsies are routinely scored using the Oxford classification of IgAN (MEST-C score) (16). In this study, we focused on the “T” score, which defines the percentage of cortical area from needle biopsies affected by either tubular atrophy or interstitial fibrosis (whichever is greater) as T0 (0–25%), T1 (26–50%), and T2 (>50%). We also used biopsies reported as normal from 13 healthy kidney transplant donors. Patients treated with corticosteroids or cancer patients in chemotherapy were excluded. Ethical approval for the use of biopsies was obtained from the Northamptonshire, Leicestershire, and Rutland Ethics Committee (UHL 09873). Needle biopsies were fixed in 10% formalin for 24 h before automated paraffin block embedding. Paraffin blocks were sectioned on a Leica microtome at 10 μm per section. Sections were treated using acid-based Ag retrieval and dewaxing solution (Abcam) at 98°C for 1 h. Sections were thoroughly washed, blocked in 10% normal donkey serum (Dako) for 1 h, and subsequently incubated with anti-human primary Abs against the following proteins: ADAMTS5 (raised in rabbit), CD64, and vimentin (mouse) from Abcam and TIMP3 (mouse), myeloperoxidase, TIMP1, lumican (LUM), ADAMTS1, versican (VCAN), collagen-4, and CD206 (all goat) from Bio-Techne. All Abs were used at 1/100 dilution for 16 h at 4°C. Sections were washed four times in PBS and incubated with fluorescent secondary Abs. Anti-rabbit 568 nm (red), anti-mouse 647 nm (blue,) and anti-goat 488 nm (green) were used. Sections were washed again four times in PBS and were covered in Vectashield antifade mounting solution before application of coverslips. Finally staining was visualized in a Zeiss Axioscope microscope using a TissueFaxs stage and image scanning multispectral software (TissueGnostics). Analysis of immunofluorescence images was performed with ImageJ. For ADAMTS5 quantitation, we used simple particle counting on the entire cortical area of ADAMTS5-stained images (renal needle biopsies sample almost exclusively the cortex). The ADAMTS5 (568 nm; red) channel was changed to black and white. We then used particle threshold 70 for all stained sections to take into account possible nonspecifically stained spots. Selecting the same threshold for all images was chosen to ensure the same level of particle bias for all stained sections. Particles were then counted automatically and raw particle numbers were divided by tissue area to correct numbers of particles to stained area size. Statistical estimation of ADAMTS5 staining was performed with two-tailed *t* test in Prism (v7). ADAMTS5 staining was also correlated against patient estimated glomerular filtration rate (eGFR) and proteinuria values using nonparametric Spearman correlation (Prism v7) and was also compared in patients separated according to their MEST-C (T) score as described above. For more than two groups statistical evaluation was performed using ANOVA followed by pairwise comparisons using Fisher least significant difference test.

### Gel electrophoresis and immunoblotting

Immunoblotting against ADAMTS5 was performed on lysates from 1 million cells (mesangial, proximal tubule epithelial cells [PTEC], leukocytes) and rat kidney tissue extracts as described in relevant sections. Cells and kidney tissue were lysed in 0.2% SDS containing broad proteinase inhibitor mixture (Sigma-Aldrich) and EDTA, protein concentration was estimated (A280 nm) and samples were mixed with standard Laemli sample buffer containing 50 mM DTT. Twenty micrograms of protein per sample were loaded on 4–12% NuPAGE mini-gels (Thermo Fisher Scientific), proteins were transferred on nitrocellulose membranes, blocked in 5% BSA for 1 h, and incubated with anti-human Ag primary Abs against what follows: ADAMTS5, VCAN (DPEAAE), biglycan, fibronectin (FN1), and complement C3 (all rabbit) from Abcam. We also used LUM (goat), osteoglycin, and decorin (rabbit) from Santa Cruz Biotechnology. Collagen-4 and myeloperoxidase (both goat) were from Bio-Techne. Complement C3C/C3D Abs (rabbit) from Dako. All Abs were diluted in 5% BSA at 2 μg/ml and membranes were incubated for 16 h at 4°C. Membranes were washed four times in PBS-Tween and incubated with HRP-conjugated swine anti-rabbit, rabbit anti-mouse, or rabbit anti-goat Abs (1/2000; Dako) for 1 h at room temperature. Membranes were washed again with PBS-Tween and exposed to ECL-prime developing reagent (Amersham). Finally, signals were visualized in a Bio-Rad Imaging System. β-actin served as a loading control in immunoblots probed with anti β-actin Abs raised in mouse (Abcam).

### Purification of total IgA from IgAN human serum using jacalin

IgA was isolated from human IgAN serum using jacalin agarose under sterile and LPS-free conditions. Jacalin is the standard total IgA purification methodology and has been used for more than 30 y (17). One milliliter human serum from IgAN patients was filtered and added to a sterile 1-ml (packed) jacalin column and left to gravity-flow for 1 h. The column was then centrifuged to remove all residual serum and washed eight times with sterile PBS. Jacalin-bound IgA was then eluted using 1 ml of 20% galactose solution (MP-Biomedicals). The column was centrifuged to collect all residual galactose-IgA solution and either used immediately or frozen at −20°C for later use. To reduce variability, for the stimulation of leukocytes and mesangial cells (see later), we used three separate pools of jacalin-purified IgA from 16 human IgAN patient serum samples in total. On cells, purified IgA was used at 200 μg/ml in serum-free DMEM.

### Complement activation assay

The Wieslab Complement System Screen (COMPL-300-RUO) was used to measure classical, alternative, and lectin complement activity present in IgA purified using jacalin from human serum. Jacalin-purified IgA from several IgAN patients was first incubated with or without ADAMTS5 (4 μg/ml, 18 h, 37°C). Following ADAMTS5 treatment, samples were diluted 1/10 in specific complement pathway diluents, and the assay was performed using kit instructions and supplied reagents and standards in duplicate. One hundred microliters of samples, blanks, controls were incubated for 70 min at 37°C. After incubation, wells were washed three times and then incubated with conjugate solutions for 30 min at 20°C. After incubation, the wells were washed and substrate solution was added and left for 30 min at 20°C to develop. The reaction was stopped using 5 mM EDTA, and the plate was read at 405 nm. Results were quantified as per kit instructions; subtract the absorbance of the blank from samples/controls, calculate the mean OD405 nm values and then the percent complement activity as follows: (Sample – NC)/(PC – NC) × 100. There were separate positive controls for the three different pathways. Whereas the classical and lectin pathways returned low levels of activity in comparison with positive controls, alternative pathway activity was undetectable.

### Cultured human mesangial and PTEC

Primary human mesangial cells from healthy kidneys and immortalized human (HK2 type) were obtained from American Type Culture Collection. Mesangial cells were used between passages 8 and 10, whereas PTECs were at passage 16–18. Both cell types were cultured and maintained in high-glucose DMEM and 10% ultra-low Ig FCS (Thermo Fisher Scientific) in 24-well plates (∼1,000,000 cells per well, 100% confluent). Before stimulation, cells were serum-starved for 6 h in serum-free DMEM (Sigma-Aldrich). Cells were stimulated in 250 μl of serum-free DMEM for different periods and with different factors as indicated. Following stimulation periods, the serum-free culture medium was collected, cells were washed twice with PBS and lysed with 0.2% SDS (with proteinase inhibitor mixture) for immunoblotting.

### Cultured human leukocytes

Human leukocytes were isolated from the blood of healthy donors using two 5 min rounds of erythrocyte lysis (erythrocyte lysis buffer; Roche Diagonistics) and five washes and spins with RPMI 1640 supplemented with 2% ultra-low Ig FCS (Thermo Fisher Scientific). Five hundred thousand leukocytes were plated per well of a 48-well plate. Cells were stimulated with 100 ng/ml rIL-1B, IL-10, IFNG (PeproTech), 1 μg/ml LPS (Sigma-Aldrich), or 200 μg/ml IgAN serum jacalin-purified total IgA for 24 h. Following stimulation, leukocytes were spun and washed two times with PBS. Leukocyte pellets were finally lysed with 0.2% SDS (with proteinase inhibitor mixture) and were used for immunoblotting as indicated. The leukocyte culture medium was used for ADAMTS5 ELISA (Abbexa) at 1/100 dilution using kit instructions and supplied reagents and standards in duplicate.

### ADAMTS5 proteolysis

For ADAMTS5 in vitro tissue digestion, kidneys from euthanized healthy adult rats, were snap-frozen to stop cellular metabolic activity, diced into smaller tissue explants and then washed four times in PBS to remove blood contamination. Forty milligrams of diced kidney explants were incubated either in 400 μl of plain enzyme digestion buffer (control) or digestion buffer supplemented with 4 μg/ml ADAMTS5 (Bio-Techne) for 36 h at 37°C. For comparison in immunoblots (see relevant *Results*), diced kidney explants were also incubated in digestion buffer with 4 μg/ml of either ADAMTS1 or ADAMTS4, for 36 h at 37°C (both enzymes from Bio-Techne). ADAMTS5 digestion buffer was 50 mM Tris, 100 mM NaCl, 5 mM CaCl_2_, and 0.05% Tween-20 (pH 7.5) as per the vendor’s instructions. For enzymatic digestion of jacalin-purified IgA and associated proteins, purified IgA was mixed with 2 μg/ml ADAMTS5 in digestion buffer for either 36 or 18 h (as indicated) at 37°C. For treatment of primary cells with ADAMTS5 in culture, cells were incubated with 2 μg/ml ADAMTS5, which was diluted in sterile PBS rather than digestion buffer. Importantly, the catabolic activity of ADAMTS5 in cell culture might not be optimal as the enzyme operates at maximum efficiency in the presence of Tween-20 (see ADAMTS5 digestion buffer below), which was not included in culture because the detergent might affect the cells. In addition, in cell culture conditions the enzyme is susceptible to cellular metabolism and likely interference by natural metalloproteinase inhibitors (TIMPs) that might be produced by cells in culture.

### Proteomics analysis of kidney treated with ADAMTS5

For proteomics and immunobloting analysis, control and ADAMTS5-treated rat kidneys, were extracted with 0.2% SDS, supplemented with proteinase inhibitors (P8340; Sigma-Aldrich) 10 mM EDTA, using vigorous shaking for 5 h. Low concentration SDS was used to solubilize easily extractable extracellular and cellular proteins and to avoid generic extraction of the kidney matrix by stronger detergents or denaturing buffers. Extracts were spun to clarify and protein concentration was estimated using A280. Control and ADAMTS5-treated, 0.2% SDS kidney extracts (see above) were processed using the iST Kit (PreOmics) to remove salts and detergents before liquid chromatography–tandem mass spectrometry (LC-MS/MS). iST-processed extracts were lyophilized, and each sample was dissolved in 45 μl of 100 mM triethylammonium bicarbonate buffer, reduced with 2 mM TCEP (tris-2-carboxyethyl-phosphine) and alkylated with 15 mM chloroacetamide. Protein digestion was performed with porcine trypsin (100 ng/μl in 10 mM HCl) enhanced in a microwave instrument (Discover System) for 30 min at 5 W and 60°C. Samples were dried and dissolved in 20 μl of 0.1% formic acid for LC-MS/MS analysis. Three microliters (2 μg) protein of rat kidney extract (per sample) was injected on a nanoAcquity Ultra Performance Liquid Chromatography (Waters) connected to a Q-Exactive mass spectrometer (Thermo Fisher Scientific) equipped with a Digital PicoView source (New Objective). Solvent composition at the two channels was 0.1% formic acid for channel A and 0.1% formic acid, 99.9% acetonitrile for channel B. Peptides were trapped on a Symmetry (Waters) C18 trap-column (5 μm, 180 μm × 20 mm) and separated on a BEH300 (Waters) C18 column (1.7 μm, 75 μm × 150 μm) at a flow rate of 250 nl/min using a gradient from 1 to 40% acetonitrile in 90 min. The mass spectrometer was operated in data-dependent mode (DDA), acquiring full-scan mass spectrometry spectra (350–1500 m/z) at a resolution of 70,000 at 200 m/z (mass-to-charge) after accumulation to a target value of 3,000,000, followed by high-energy collision dissociation fragmentation on the 12 most intense signals per mass spectrometry cycle. high-energy collision dissociation spectra were acquired at a resolution of 35,000 using normalized collision energy of 25 and a maximum injection time of 120 ms. Automatic gain control was set to 50,000 ions. Charge state screening was enabled. Singly and unassigned charge states were rejected. Only precursors with intensity above 8300 were selected for tandem mass spectrometry (MS/MS) (2% underfill ratio). Precursor masses previously selected for MS/MS were excluded from further selection for 30 s, and the exclusion window was set at 10 ppm. The samples were acquired using internal lock-mass calibration on m/z 371.1010 and 445.1200. Acquired raw MS/MS files were converted to Mascot Generic Format (.mgf) using ProteoWizard (http://proteowizard.sourceforge.net/) and proteins were identified using the Mascot search engine (Matrix Science, version 2.5.1.3.) searching data against the Swissprot human database (version 2018-07-18). Carbamidomethylation of cysteine was set as fixed modification, whereas methionine oxidation was set as variable. Enzyme specificity was set to trypsin, allowing a maximum of two missed cleavages. Precursor and fragment tolerance was set to 0.03 Da and 10 ppm, respectively. The Mascot.dat file results were loaded in Scaffold (Proteome Software) to validate peptide and protein identifications, to visualize and count peptide and spectra identifications. The maximum false-discovery rate was set to 0.01 for peptides and 0.05 for proteins. Only proteins identified with at least two peptides were considered for follow up analysis. Raw MS/MS files are provided online at Mendeley Data (https://data.mendeley.com/datasets/5vx85p9fjw/draft?a=fc5c7b15-55dc-479c-b1ec-9b1d3001a4b4). Protein-protein interaction network analysis of genes and proteins was performed using StringDB v11 and Cytoscape (v3.6.1). BinGO was used in Cytoscape to identify gene ontology (GO) annotations. Statistical estimation of ADAMTS5-affected proteins was performed using *t* test on spectral counts in Scaffold. Volcano plots were generated in Prism (v7) using *t* test *p* value (from 0.05 to 0.0000001) and spectral counts fold-change (−20 to +20). Heat maps were generated on top-50 significantly affected proteins as described in *Results*.

## Results

### ADAMTS5 is increased in human IgAN biopsies

**FIGURE 1.**
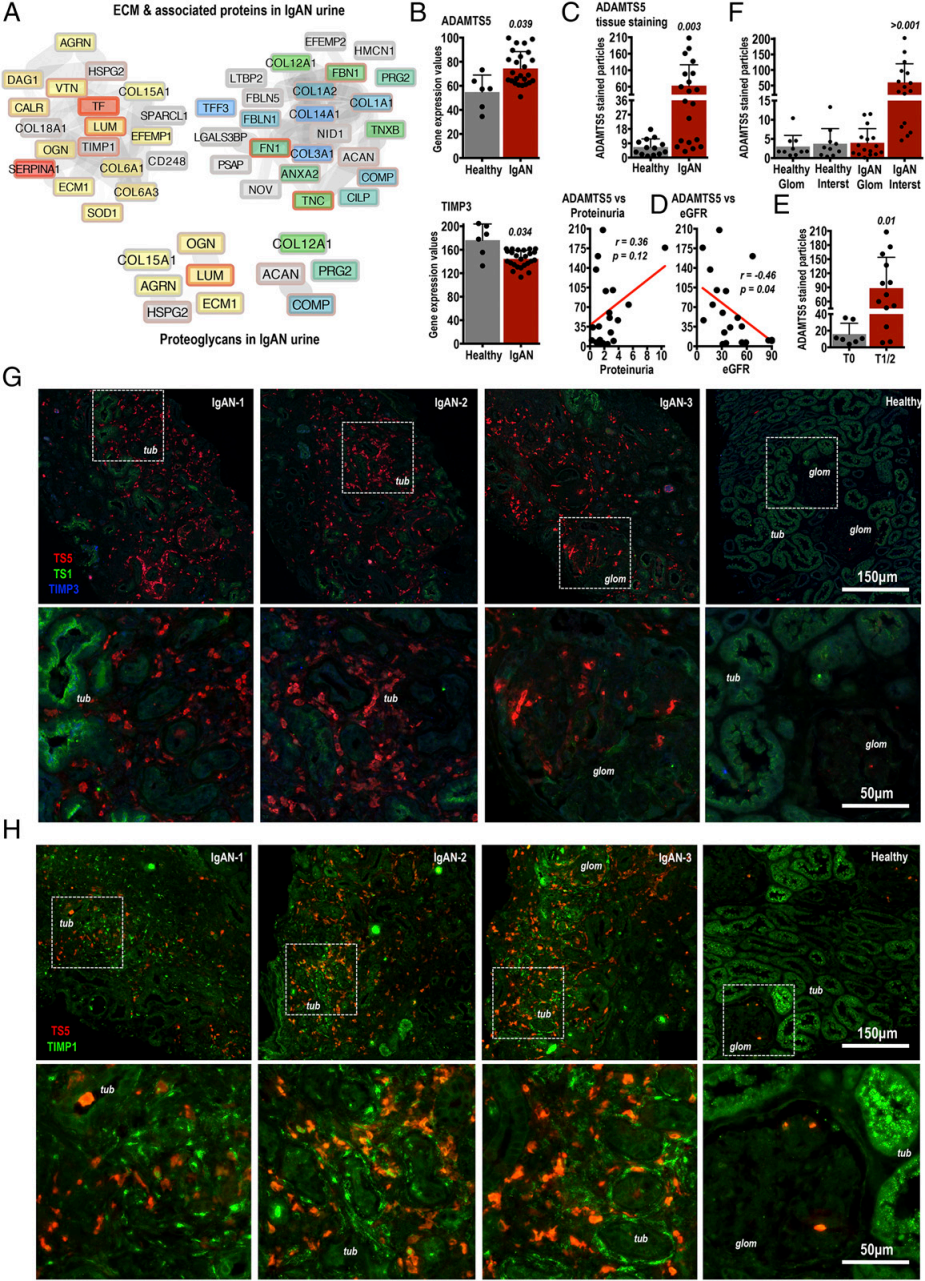
Identification of matrix proteins by urine proteomics leads to the discovery of ADAMTS5 in IgAN kidneys. (**A**) LC-MS/MS proteomics of healthy donor and IgAN patient urine identified multiple ECM proteins present in IgAN urine (see Ref. 13). Matrix proteins were selected using gene ontology (BinGO) and are presented as a protein-protein interaction network. Yellow and red proteins are increased in IgAN urine, gray are unchanged, and blue are decreased in comparison with healthy samples. Proteomics analysis including all identified proteins is described in detail in (13). (**B**) The mRNA expression of ADAMTS5 and TIMP3 was examined in a published microarray dataset comparing 25 IgAN patients with six healthy donor kidney biopsies. Microarray gene expression was retrieved directly from National Center for Biotechnology Information–Gene Expression Omnibus (GSE35488; https://www.ncbi.nlm.nih.gov/geo/query/acc.cgi?acc=GSE35488). Microarray data are available in Supplemental Table I. ADAMTS5 and TIMP3 were significantly regulated in IgAN (Supplemental Table I). Differences were examined using standard *t* test using data curated and processed by NCBI-GEO as described in GSE35488; https://www.ncbi.nlm.nih.gov/geo/query/acc.cgi?acc=GSE35488. The *p* values are shown. (**C**) ADAMTS5 protein immunofluorescence was measured in 13 healthy (kidney transplant donors) versus 20 IgAN patients. IgAN donors were biopsy diagnosed. ADAMTS5 staining was quantified using ADAMTS5-stained particle counting corrected to biopsy tissue area on ImageJ and values were compared using two-tailed *t* test. (**D**) For the 20 IgAN patients, ADAMTS5 immunofluorescence was correlated to their matching urine protein concentration (proteinuria; mg/dl) and eGFR (estimated glomerular filtration rate; ml/min/1.73 m^2^) values. The *r* and *p* values were computed using nonparametric Spearman test given the dissimilar nature of the correlated values. (**E**) ADAMTS5 staining was also compared in the same IgAN biopsies but biopsies were grouped according to their histological MEST-C score. We focused on the (T) score (percentage of biopsy area affected by tubular atrophy or interstitial fibrosis, whichever is greater) and compared less affected T0 biopsies (*n* = 7) with more affected T1/T2 biopsies (*n* = 13). There was a significant increase in ADAMTS5 staining in T1/T2 specimens (*t* test). (**F**) ADAMTS5 biopsy staining was also measured separately in the tubulointerstitium and glomeruli and compared in healthy versus IgAN biopsies. ADAMTS5 staining in the IgAN interstitium (*IgAN Interst*) is significantly increased in comparison with staining in IgAN glomeruli (*IgAN Glom*), as well as in comparison with healthy interstitium and glomeruli, which are NS different to each other (ANOVA with Fisher least significant difference, multiple comparison test). (**G** and **H**) Examples of ADAMTS5 immunofluorescence in IgAN and healthy kidney biopsies costained with relevant proteins. In (G) ADAMTS5 (red) was costained with ADAMTS1 (green) and TIMP3 (blue). TIMP3 staining was below the threshold of confident detection in either IgAN or healthy biopsies. In (H) ADAMTS5 (red) was costained with TIMP1 (green). ADAMTS5 is close but does not appear to colocalise with TIMP1 in affected tubulointerstitial areas. *tub* denotes renal tubules and *glom* denotes glomeruli. Note that ADAMTS5 is increased in areas of tubulointerstitial inflammatory infiltration and remodelling. IgAN glomeruli also contain ADAMTS5^+^ cells. In healthy biopsies, there were sporadic ADAMTS5-stained (red) cells in the interstitium [(G) healthy] and glomeruli [(H) healthy]. White-dashed boxes denote areas shown in higher magnification in lower panels.

Recent proteomics analysis of urine collected from healthy donors and IgAN patients identified multiple extracellular proteins in urine (13). Matrix proteins identified in urine (13) are summarized as protein-protein interaction networks in Fig. 1A. Our attention was captured by tissue proteoglycans aggrecan (ACAN), LUM, mimecan (OGN; osteoglycin), collagen-12 (COL12A1), collagen-15 (COL15A1), PRG2 and ECM1 as well as heparan sulfate proteoglycans agrin (AGRN) and HSPG2 (Fig. 1A). Given that many of these matrix proteins have not been studied in IgAN, we queried whether their expression in IgAN kidneys is altered at the transcript level. To this end, we examined a published microarray dataset (GSE35488 Gene Expression Omnibus–National Center for Biotechonolgy Information; https://www.ncbi.nlm.nih.gov/geo/query/acc.cgi?acc=GSE35488) comparing tubulointerstitial transcript expression of healthy versus IgAN human kidney biopsies (18). The GSE35488 dataset is also downloaded in Supplemental Table I. While the matrix proteins that we identified in urine by proteomics (Fig. 1A) were not differentially regulated at the transcript level in the GSE35488 IgAN microarray, we noticed a modest increase in the mRNA expression of the glycoprotein-degrading metalloproteinase ADAMTS5 in IgAN, coupled by a small downregulation of its natural inhibitor TIMP3 (Fig. 1B). For comparison, ADAMTS4 and ADAMTS1, family enzymes with similar and overlapping matrix substrate specificity, were not regulated (Supplemental Table I). In fact, excluding ADAMTS5, no other metalloproteinase transcripts (from MMP1 to MMP28 and ADAMTS1 to ADAMTS13) were significantly regulated in the GSE35488 microarray (Supplemental Table I). These findings should be treated with statistical caution given the large difference in sample size between healthy (*n* = 6) and IgAN (*n* = 25) biopsies used for the GSE35488 microarray (18) and as a result, the weak statistical differences observed.

The expression and function of ADAMTS5 in IgAN or kidney disease is unknown. To this end, we performed immunostaining on healthy (kidney transplant donors) versus IgAN kidney biopsies from patients with clinically proven IgAN. The semiquantitative analysis of all ADAMTS5 immunostaining performed is summarized in Fig. 1C (number of ADAMTS5-stained particles corrected to biopsy surface area). Overall, there was an increase in ADAMTS5 immunostaining in IgAN biopsies (*p* = 0.003), but also a clear difference in ADAMTS5 levels between IgAN patients. Thirteen IgAN biopsies had high ADAMTS5, whereas there were seven biopsies with staining levels comparable to controls (Fig. 1C). When we compared ADAMTS5 biopsy staining with urine protein levels (proteinuria) and eGFR for the same IgAN patients (Fig. 1D), we noted a positive but NS correlation between ADAMTS5 staining and proteinuria (*r* = +0.36; *p* = 0.12) and a marginally significant negative correlation with eGFR (*r* = −0.46; *p* = 0.04) indicating that ADAMTS5 might be increased as kidney function declines in IgAN, causing reduction in glomerular filtration (eGFR) and increase in urine protein concentration (Fig. 1D). We then compared ADAMTS5 staining by splitting IgAN biopsies according to their histological MEST-C score (Fig. 1E). MEST-C score (or Oxford classification of IgAN) is used routinely in the clinical setting to classify the pathological changes observed in IgAN kidney biopsies (16). The MEST-C score defines the percentage of biopsy cortical area affected by tubular atrophy or interstitial fibrosis (T) as T0 (0–25%), T1 (26–50%), and T2 (>50%). Biopsies classified as T1/T2 had more ADAMTS5 staining in comparison with less affected T0 biopsies (Fig. 1E), suggesting that the enzyme might be linked to increased tissue pathology. Finally, we compared ADAMTS5 staining in biopsies separately in tubulointerstitial areas and glomeruli (Fig. 1F). Most ADAMTS5 staining in IgAN biopsies is in the interstitium (∼90% of staining) consistent with tubulointerstitial inflammation. Instead, in healthy biopsies there is an even distribution in ADAMTS5 staining in the glomeruli and interstitium (Fig. 1F).

In IgAN biopsies, high ADAMTS5 expression was associated with presumably cellular infiltrates decorating tubulointerstitial areas (Fig. 1G, 1H, red). ADAMTS5 staining was also seen in IgAN glomeruli (Fig. 1G; IgAN-3). Few sporadic ADAMTS5^+^ cells were present in healthy subjects (Fig. 1G, 1H; healthy). In contrast to ADAMTS5, ADAMTS1 (Fig. 1G; green) was in the luminal surface of tubules in both IgAN and healthy subjects, but was likely not expressed by ADAMTS5^+^ cells. The natural ADAMTS inhibitor TIMP3 (Fig. 1G; blue), which was downregulated in the GSE35488 microarray (Fig. 1B, Supplemental Table I) was very weakly stained (see Fig. 1G; IgAN-1) and we were unable to detect it reliably in either IgAN or healthy specimens. TIMP1 (Fig. 1H; green), the other classic metalloproteinase inhibitor associated with inhibition of MMPs but not ADAMTSs, was in close proximity to ADAMTS5 (Fig. 1H; red) in IgAN lesions but without absolute colocalisation. Notably, TIMP1 was identified in IgAN urine by proteomics (Fig. 1A).

### ADAMTS5 is expressed by CD64^+^ cells in IgAN lesions

**FIGURE 2.**
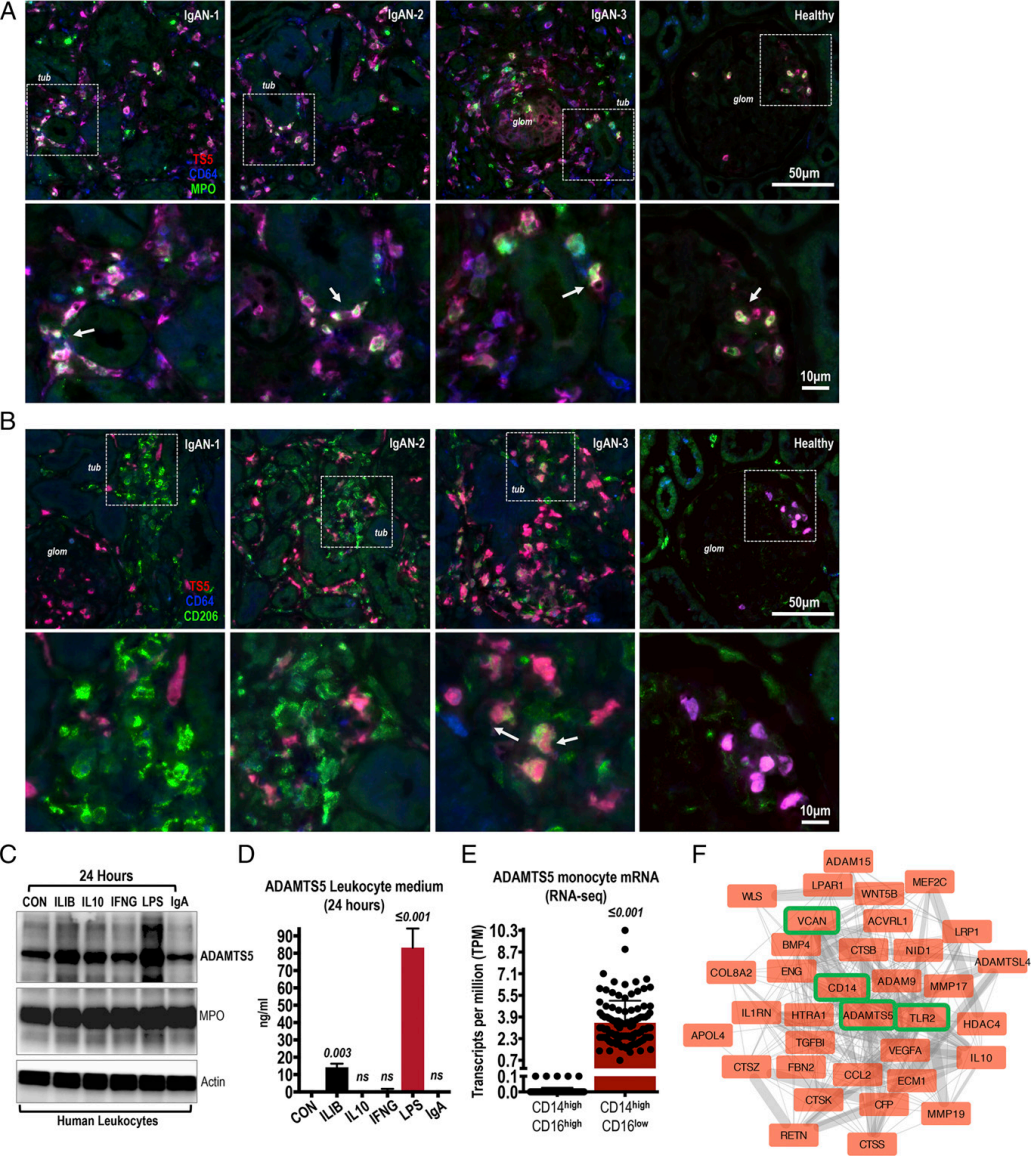
Costaining of ADAMTS5 in IgAN biopsies with inflammatory cell markers. (**A**) In IgAN and healthy kidney biopsies, ADAMTS5 (red) was costained with CD64 (blue) and myeloperoxidase (MPO; green). ADAMTS5 colocalises with CD64^+^ cells in both IgAN and healthy biopsies (cells with ADAMTS5 and CD64 colocalisation appear as purple). There are also MPO^+^ cells and sporadic triple-positive ADAMTS5-CD64-MPO (white) cells were present indicating expression of ADAMST5 by infiltrating monocytes and neutrophils (see arrows). Note intense accumulation of ADAMTS5^+^-CD64^+^-MPO^+^ cells in the interstitium around tubules (*tub*) in affected IgAN specimens. In healthy biopsies, triple-stained cells were mainly seen in glomeruli (*glom*), presumably naturally perfusing circulating leukocytes. (**B**) ADAMTS5 (red) was costained with CD206 (green) and CD64 (blue). ADAMTS5 colocalises with CD64 (purple cells). In contrast, colocalisation of ADAMTS5 (red) with interstitial CD206 (green) is weak and sporadic in inflamed areas (see IgAN-3; arrows) indicating different cellular origins. (**C**) ADAMTS5 was immunoblotted on leukocyte lysates. Leukocytes were isolated from healthy donors by erythrocyte lysis and were cultured for 24 h with 100 ng/ml rIL-1B, IL-10, IFNG, 1 μg/ml LPS, or 200 μg/ml IgAN-serum jacalin-purified IgA. Myeloperoxidase was used for comparison and βActin as a loading control. (**D**) ADAMTS5 protein levels measured by ELISA in the culture medium of leukocytes stimulated for 24 h as in (C). (**E**) RNA sequencing expression of ADAMTS5 in classical versus nonclassical monocytes. Data retrieved from the Human Protein Atlas and (26). (**F**) ADAMTS5 first-degree neighbors (proteins interacting directly with ADAMTS5 via a single edge) derived from the classical monocyte elevated gene interactome. Classical monocyte elevated genes were downloaded from the Human Protein Atlas. Protein-protein interactions were processed and visualized in Cytoscape.

The size of ADAMTS5 staining clusters suggested expression of the enzyme by infiltrating cells in inflamed IgAN kidneys. First, ADAMTS5 (Fig. 2A; red) colocalized with the monocyte (and neutrophil) Fcγ receptor CD64 (Fig. 2A; blue) and sporadically with myeloperoxidase (Fig. 2A; MPO green), expressed by activated monocytes and granulocytes (19). IgAN infiltrates in Fig. 2A coexpress ADAMTS5 (red) and CD64 (blue) and we also spotted few triple-positive ADAMTS5^+^-MPO^+^-CD64^+^ cells (highlighted in Fig. 2A; arrows). ADAMTS5^+^ cells found in healthy glomeruli were also MPO^+^ and CD64^+^ (Fig. 2A; healthy) presumably naturally trafficking leukocytes. Second, ADAMTS5 (Fig. 2B; red) showed limited association with the mannose receptor CD206 (Fig. 2B; green), a surface protein expressed in macrophages with a likely preference for tissue-resident macrophages (20). ADAMTS5 did not colocalize with most CD206^+^ cells in IgAN tubulointerstitium and glomeruli, and we found few sporadic triple-positive ADAMTS5^+^-CD206^+^-CD64^+^ cells (Fig. 2B; IgAN-3 arrows).

We also costained ADAMTS5 with classic cellular filament proteins vimentin and smooth muscle actin (Supplemental Fig. 1). ADAMTS5^+^ infiltrates appear to express vimentin (Supplemental Fig. 1A; see IgAN-1 mild purple color). This is expected given that vimentin is expressed by monocytes and granulocytes (21). In contrast, ADAMTS5 did not colocalize with vimentin^+^ cells in glomeruli (Supplemental Fig. 1A; IgAN-3). In healthy kidney glomeruli, vimentin is preferably expressed by mesangial cells, whereas podocytes are also known to express vimentin and other intermediate filaments (22), especially in disease (23). Similarly, ADAMTS5 did not appear to colocalize with scattered vimentin^+^ cells in the fibrotic IgAN tubulointerstitium (Supplemental Fig. 1A; IgAN-2 *fibr*; note highlighted area with only blue vimentin^+^ cells), likely tubule epithelial cells undergoing mesenchymal transformation to vimentin^+^ myofibroblasts (24). ADAMTS5 did not colocalize with IgA (Supplemental Fig. 1A; green) indicating lack of association between the enzyme and the prime pathogenic factor of IgAN. As for vimentin, smooth muscle actin, a classic marker of smooth muscle cells and myofibroblasts, did not associate with ADAMTS5, indicating that fibrotic cells are probably not related to ADAMTS5 expression in IgAN kidneys (Supplemental Fig. 1B; smooth muscle actin green).

Given the likely association of ADAMTS5 with inflammatory infiltrates, the expression of ADAMTS5 was examined in cultured human leukocytes (Fig. 2C, 2D). High concentration LPS stimulation for 24 h caused an increase in the expression and secretion of ADAMTS5, whereas IL-1B had a more modest effect (Fig. 2C, 2D). Total IgA, purified from the serum of IgAN patients using jacalin, did not affect ADAMTS5 levels suggesting that circulating IgA might not be related to ADAMTS5 expression in leukocytes at least in vitro (Fig. 2C, 2D). The induction of ADAMTS5 in leukocytes was interesting given that expression and function of ADAMTS5 in myeloid cells has not been studied. To investigate further, we analyzed recently published myeloid cell RNA-sequencing datasets examining mRNA expression in all different myeloid cell types from multiple donors (25–27). This data are recently available via the Human Protein Atlas (https://www.proteinatlas.org/ENSG00000154736-ADAMTS5/blood/monocytes#schmiedel_classical_monocyte). To our surprise, ADAMTS5 is robustly enriched specifically in classical monocytes (CD14^high^/CD16^low^) in comparison with every other myeloid cell type (Fig. 2E; the graph depicts a comparison of classical CD14^high^/CD16^low^ versus nonclassical CD14^high^/CD16^high^ monocytes). In fact, according to the blood atlas, ADAMTS5 is one of just 18 classical monocyte-enriched genes (classical monocyte-enriched genes). We then used simple protein-protein interaction network mapping of ADAMTS5 first-neighbor classical monocyte-enriched genes, thus genes directly associated with ADAMTS5 from the classical monocyte-elevated gene signature via a single edge (Fig. 2F). Together with multiple interesting inflammatory proteins, we noted that the ADAMTS5 monocyte expression neighborhood contains the ADAMTS5 substrate and monocyte protein VCAN as well as the classic pattern-recognition receptor TLR2 and its adaptor CD14, both typical monocyte markers. Interestingly, VCAN fragments might activate TLR2 (28), indicating a possible immunoregulatory involvement of ADAMTS5 in monocytes via VCAN and TLR2. Notably, ADAMTS5^+^ inflammatory infiltrates were not unique to IgAN as the enzyme was also present in membranous nephropathy biopsies (Supplemental Fig. 2A) and focal segmental glomerulosclerosis lesions (Supplemental Fig. 2B). Thus, ADAMTS5 is associated with kidney inflammation and is not exclusively expressed in IgAN lesions.

### ADAMTS5 is associated with matrix proteins in IgAN lesions

**FIGURE 3.**
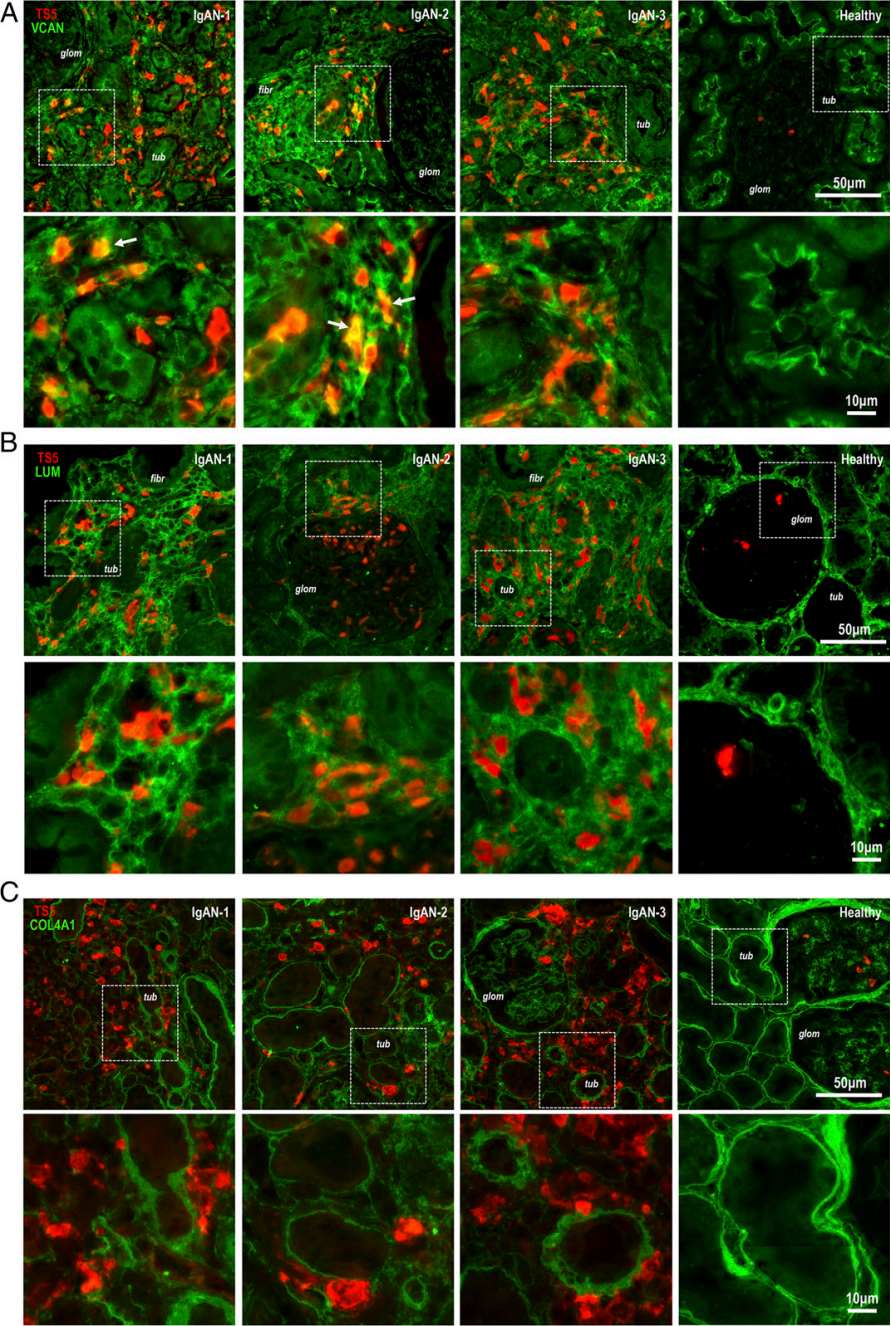
Costaining of ADAMTS5 in IgAN biopsies with extracellular matrix proteins. (**A**) In IgAN and healthy kidney biopsies ADAMTS5 (red) was costained with VCAN (green). In the IgAN tubulointerstitium (*tub*), there is diffuse and disorganised VCAN staining presumably indicating fibrotic remodelling (see IgAN-2; *fibr*). VCAN was mainly observed interstitially, whereas glomeruli (*glom*) were almost completely devoid of the large proteoglycan (see IgAN-1 and IgAN-2; *glom*). ADAMTS5 is closely related to VCAN staining but only few sporadic areas of colocalisation (see arrows IgAN-1 and IgAN-2). In healthy biopsies, VCAN is almost exclusively found in the luminal surface of renal tubules (*tub*). (**B**) ADAMTS5 (red) was costained with LUM (green). In IgAN biopsies, the fibrotic (*fibr*) interstitial matrix contains LUM. ADAMTS5^+^ cells are embedded within areas of LUM accumulation but there is no colocalisation. Similar to VCAN, glomeruli are devoid of LUM. In healthy biopsies, LUM is brightly staining the intact peritubular matrix. (**C**) Finally, ADAMTS5 (red) was costained with basement membrane proteoglycan collagen-4 (COL4A1; green). The Ab is specific for the α-1 chain. There is disorganization of peritubular collagen-4 in the IgAN tubulointerstitium (see IgAN-1, IgAN-2, and IgAN-3). Interstitial areas of collagen-4 remodelling are decorated with ADAMTS5^+^ cells in IgAN biopsies. Bright peritubular and periglomerular collagen-4 staining is seen in healthy biopsies. For (A)–(C) stains, white dashed boxes denote areas that are shown in higher magnification in the lower panels.

Although the exact substrate specificity of ADAMTS5 in different tissues is not fully known, previous work has shown that ADAMTS5 cleaves chondroitin-sulfate proteoglycans aggrecan, VCAN, and biglycan (14), plus a few other glycoproteins (15). ADAMTS5 (Fig. 3A; red) was first costained with VCAN (Fig. 3A; green) whose function in IgAN is unknown. In ADAMTS5^+^ IgAN lesions, the enzyme was closely related with areas of dense VCAN accumulation (Fig. 3A). In contrast, in healthy biopsies, VCAN was specifically present in the luminal surface of renal tubules (Fig. 3A; healthy). ADAMTS5 was also costained with the small keratan-sulfate proteoglycan LUM (Fig. 3B, green), which we identified in IgAN urine by proteomics (see Fig. 1A). LUM is not a known ADAMTS5 substrate and its function in the kidney is also unknown. In IgAN biopsies ADAMTS5^+^ cells were present in the meshwork of tubulointerstitial LUM accumulation (Fig. 3B). We also examined the relationship of ADAMTS5 with collagen-4 (Fig. 3C; COL4A1, green), an abundant kidney basement-membrane proteoglycan. ADAMTS5^+^ cells were associated with collagen-4 staining delimiting tubule and glomerular basement membranes (Fig. 3C). The enzyme was increased in areas of collagen-4 remodelling and irregularity (Fig. 3C). Thus, ADAMTS5 is present in areas with remodelling of matrix proteins and is likely associated with inflammatory cell infiltrates in IgAN kidneys.

### Examining ADAMTS5 kidney proteolysis with proteomics

**FIGURE 4.**
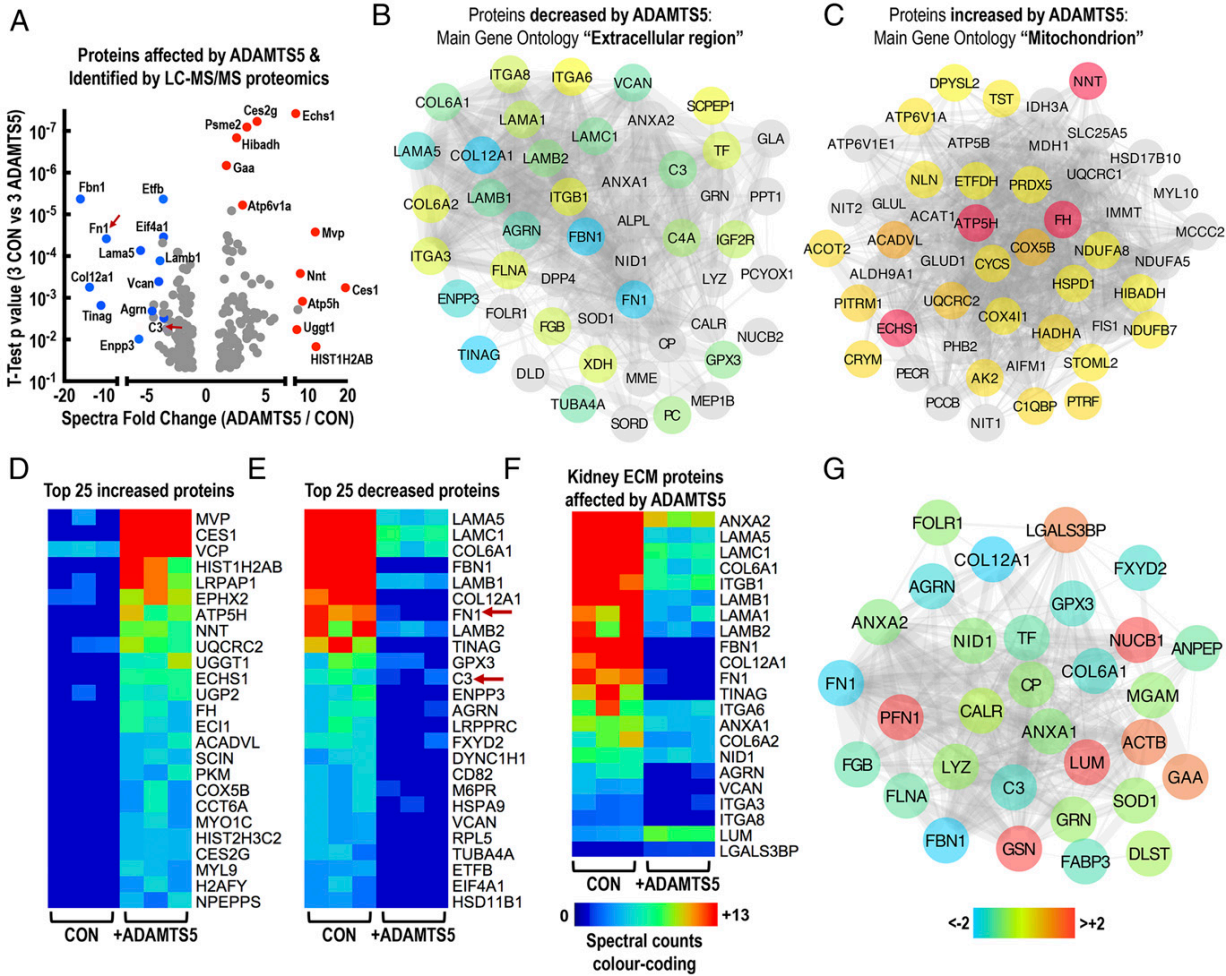
The proteolytic activity of ADAMTS5 on the kidney matrix. (**A**) The *t* test and fold-change spectral count volcano plot summarizing the effect of ADAMTS5 on the kidney proteome following LC-MS/MS analysis (spectral counts are displayed in full in Supplemental Table II). Rat kidney explants were incubated with 4 μg/ml ADAMTS5 for 36 h in enzyme digestion buffer to allow proteolysis of the kidney tissue. Control kidney explants were incubated for the same time in digestion buffer without ADAMTS5. Following incubation, proteins were extracted using 0.2% SDS and analyzed by LC-MS/MS in a Q-Exactive Orbitrap. Affected proteins were measured using spectral counting (Scaffold) and differences were estimated using *t* test (three controls versus three ADAMTS5-treated kidneys). Highly affected proteins with a significant change in spectral counts are indicated on the volcano plot. Blue dots are proteins with decreased spectral counts, whereas red dots are proteins with increased spectral counts. (**B** and **C**) The main GO of proteins with significantly altered spectral counts following ADAMTS5 treatment are depicted as protein-protein interaction networks (String and Cytoscape). (B) Proteins with significantly decreased spectral counts following ADAMTS5 treatment and main GO “Extracellular region.” (C) Proteins with significantly increased spectral counts following ADAMTS5 treatment and main GO “Mitochondrion.” (**D**–**F**) The color-coded heatmaps visualize differences in spectral counts identified by LC-MS/MS for the 25 top significantly (*t* test *p* value) increased (D) and 25 top significantly decreased (E) proteins following ADAMTS5 treatment of kidney explants (three CON versus three ADAMTS5-treated kidneys). (F) Depicts spectral counts of ECM-related kidney proteins affected by ADAMTS5. Spectral counts are displayed in Supplemental Table II. (**G**) Color-coded protein interaction network of 31 proteins affected by ADAMTS5 digestion in the kidney but also found in IgAN urine by proteomics. ADAMTS5-affected proteins can be found in IgAN urine.

To examine the possible proteolytic effect of ADAMTS5 on the renal tissue, we performed in vitro incubation of healthy rat kidney explants with ADAMTS5 in metalloproteinase digestion buffer for 36 h. Healthy rat kidney was used as a simple tissue substrate for ADAMTS5, as we were not able to access healthy, nonfixed human kidney specimens. Kidney explants were dissected and snap-frozen prior to incubation with ADAMTS5 to ensure absence of endogenous metabolic activity. The effect of ADAMTS5 on kidney proteins was then measured by shotgun LC-MS/MS proteomics of the ADAMTS5-treated and untreated (control) kidney extracts (Fig. 4A). Proteomics identified 750 proteins with stringent selection criteria (Supplemental Table II provides spectral counts and Supplemental Table III provides full MS/MS details for all identified spectra). Raw MS/MS data files are accessible via Mendeley Data (https://data.mendeley.com/datasets/5vx85p9fjw/draft?a=fc5c7b15-55dc-479c-b1ec-9b1d3001a4b4). Spectral counting was used to estimate changes in kidney proteins after ADAMTS5 incubation. (Supplemental Table II provides exact *t* test *p* values and fold-change between control and ADAMTS5-treated kidney explants). The main GO of proteins decreased by ADAMTS5 was, perhaps predictably, “Extracellular Region” (48 significantly decreased proteins; Fig. 4B) reflecting the catabolic effect of ADAMTS5 on extracellular proteins. In contrast, the main GO of proteins increased after incubation of kidney explants with ADAMTS5 was “Mitochondrion” (50 significantly increased proteins; Fig. 4C). This puzzling effect is likely explained by the fact that ADAMTS5 proteolytic activity altered tissue integrity and as a result, mitochondrial proteins were more easily solubilized from kidney explants. The 50 proteins mostly affected by ADAMTS5 (in terms of number of differential spectral counts; Supplemental Table II) are presented in Fig. 4D, 4E (4D, 25 most increased proteins; 4E, 25 most reduced kidney proteins). Differential expression analysis with spectral counts and *t* test is used in this study as a simple comparative tool to examine the effect of ADAMTS5 on kidney proteins in high throughput and is not intended for the categorical determination of enzymatic activity and substrate specificity.

ADAMTS5 affected multiple kidney ECM proteins (all affected ECM proteins are isolated in Fig. 4F); 20 matrix proteins were significantly decreased by ADAMTS5, whereas only LUM and galectin-3-binding protein (LGALS3BP) were increased (Fig. 4F), likely because their solubility increased following enzymatic digestion of other tissue proteins by ADAMTS5. Notably, the mix of ADAMTS5-affected matrix proteins was enriched in basement-membrane components including laminins (LAMA/LAMB/LAMC), collagen-6 (COL6A1/A2), collagen-12 (COL12A1), AGRN, nidogen-1 (NID1), and TINAG (tubulointerstitial nephritis Ag) (Fig. 4F). The effect of ADAMTS5 on basement-membrane proteins was accompanied by a reduction in the protein spectra of basement-membrane binding integrins ITGA3, ITGA6, and ITGA8 (Fig. 4F). ADAMTS5 also reduced VCAN, FN1, and the FN1 receptor ITGB1 (Fig. 4F). VCAN and FN1 are known ADAMTS5 targets. Thus, in vitro ADAMTS5 is effective on basement-membrane proteins, important structural components in the kidney and involved in different autoimmune pathologies (29–31). Thirty-one proteins that were significantly affected by ADAMTS5 kidney digestion were also found in human IgAN urine by proteomics (13). These are summarized in Fig. 4G and include multiple matrix glycoproteins noted above such as COL6A, LUM, NID1, FBN1, AGRN, COL12A1 as well as IgA immune complex-associated proteins FN1 and complement C3 (Fig. 4E, 4G). Thus, ADAMTS5 activity in the kidney might drive changes in the IgAN urine protein signature. Importantly, human and rat ADAMTS5 are 90% identical (UniProt) and thus it is likely that enzymatic effects are comparable between the two species. Nevertheless, the proteolytic effect of ADAMTS5 on the rat kidney described above might not be directly translatable to human kidney because of possible species differences in matrix protein expression and homology.

### ADAMTS5 proteolysis of proteins isolated with serum IgA using jacalin

The effect of ADAMTS5 on FN1 and C3 is interesting for two reasons. First, in IgAN these proteins are known to aggregate with IgA in circulating immune complexes (32–34). Second, FN1 is a known substrate of ADAMTS5 (15), whereas C3 is highly homologous to α-2-macroglobulin (A2M), a classic ADAMTS5 substrate (35) and generic proteinase inhibitor in plasma. A2M is characterized by a proteinase bait region and a reactive internal thiolester, a feature shared with C3 (36).

**FIGURE 5.**
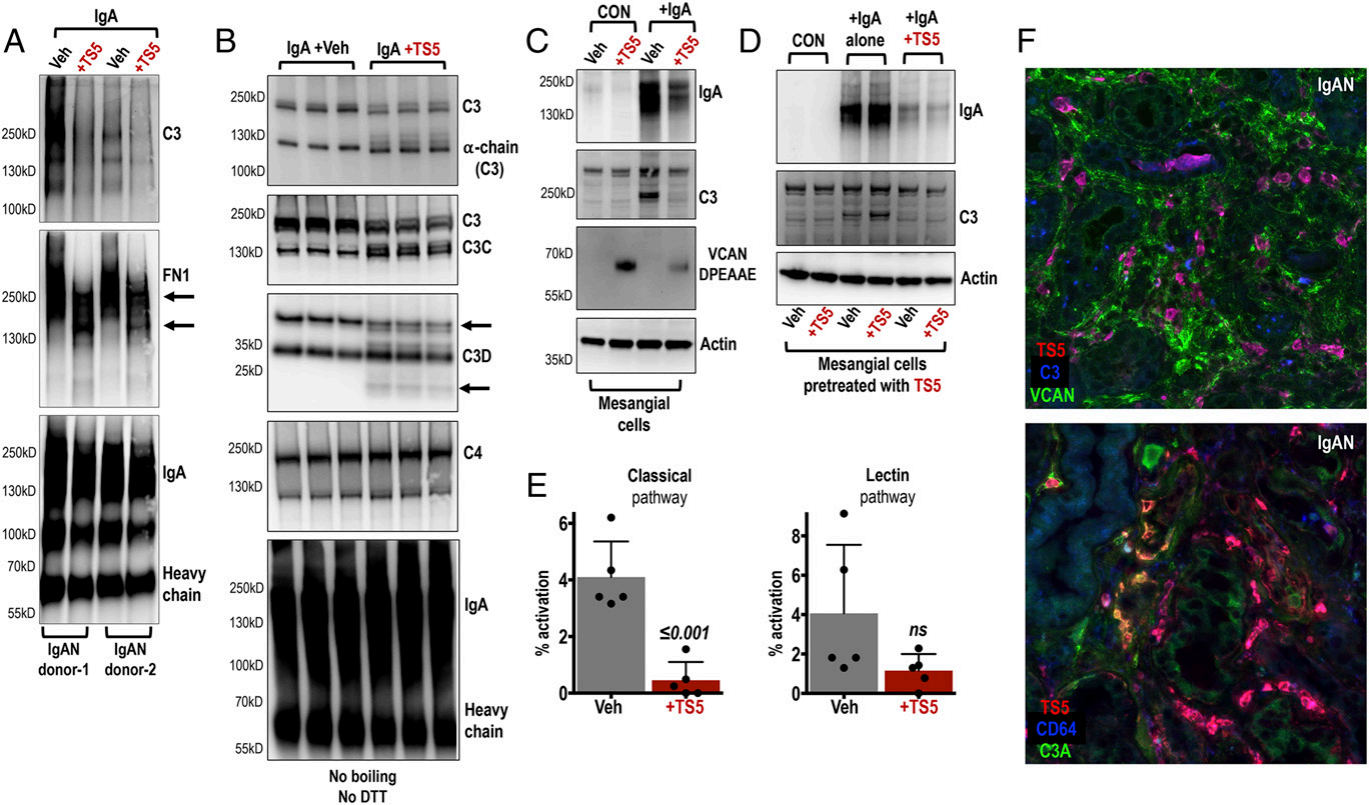
The proteolytic activity of ADAMTS5 on proteins associated with IgA purified from the serum of IgAN patients; focus on complement C3. (**A**) Immunoblotting of jacalin-purified IgA preparations from the serum of IgAN patients treated with ADAMTS5 for 36 h (+TS5). ADAMTS5 appears to fragment complement C3 and FN1 associated with the purified IgA from both donors tested. (**B**) Three separate pools of jacalin-purified IgA were treated with ADAMTS5 for 18 h (+TS5) to moderate the proteolytic effect. Samples were then immunoblotted without boiling and probed using Abs against complement C3 and C3 fragments as indicated. C4 and IgA were also probed. (**C**) Immunoblotting of primary mesangial cells incubated with IgA purified from the serum of IgAN patients for 36 h (+IgA; lanes 3 and 4). Cells were also treated for 36 h with ADAMTS5 (+TS5; lanes 2 and 4) or vehicle (veh; control). Treatment of cells with jacalin-purified IgA caused accumulation of C3 and IgA on cell layers (lanes 3 and 4). In the presence of ADAMTS5, there was a reduction in IgA and C3 immunoreactivity (lane 4). ADAMTS5 generated DPEAAE VCAN fragments on mesangial cells (lanes 2 and 4). Vehicle (veh) is sterile PBS without ADAMTS5. (**D**) Mesangial cells were pretreated for 18 h with either vehicle (veh; lanes 1, 3, 5) or ADAMTS5 (+TS5; lanes 2, 4, 6) to digest mesangial proteins. 18 h later, the pretreated cells were incubated with jacalin-purified IgA isolated from the serum of IgAN patients. The IgA samples were either plain (+IgA alone; lanes 3 and 4) or they were pretreated with ADAMTS5 for 18 h before addition onto the cells (+IgA+TS5; lanes 5 and 6). Note that predigestion of jacalin-purified IgA (lanes 5 and 6) with ADAMTS5 rather than predigestion of mesangial cells with the enzyme (lanes 4 and 6) caused a reduction in IgA and C3 immunoreactivity on lysates. (**E**) The effect of ADAMTS5 on complement activity was examined using a commercially available assay that measures the activity of classical, alternative and lectin pathways. To increase the relevance of this assay we used jacalin-purified IgA as the activating substrate as it contains IgA-associated complement proteins. Incubation with ADAMTS5 led to a significant reduction in classical pathway activity. Lectin pathway activity was reduced but inconsistent (two-tailed *t* test). Alternative pathway activity was undetectable. (**F**, top panel) Immunostaining of ADAMTS5 (red) with C3 (blue) and VCAN (green) on IgAN biopsies. ADAMTS5 colocalises with C3 in infiltrating cells. More images in Supplemental Fig. 4. (F, lower panel) Immunostaining of ADAMTS5 (red) with the anaphylatoxin C3A fragment (green) and CD64 (blue) on IgAN specimens. Colocalisation of ADAMTS5 with C3A was found in one IgAN specimen. More images in Supplemental Fig. 4.

To examine whether ADAMTS5 could digest clinically relevant FN1 and C3, we mixed ADAMTS5 with IgA purified from the serum of IgAN patients using jacalin agarose (Fig. 5A, 5B). Jacalin-purified IgA also contains aggregating FN1 and C3 (17) and is commonly used as a stimulant for mesangial cells in vitro (37). In this study, we used jacalin-purified IgA as a relevant substrate for ADAMTS5 digestion especially because of the presence of C3 and FN1 in IgA immune complexes. Incubation of jacalin-purified IgA with ADAMTS5 (36 h) caused loss of C3 immunoreactivity, whereas FN1 was fragmented by ADAMTS5 with a visible reduction in its molecular mass (Fig. 5A). We then repeated the experiment using three separate pools of jacalin-purified IgA from IgAN patients (3 pools from 16 patients were used to ensure less variability in protein composition and digestion Fig. 5B). Incubation with ADAMTS5 was reduced to 18 h to limit proteolysis, whereas proteins were electrophoresed without boiling to limit denaturation and achieve partial preservation of native protein structures (Fig. 5B). The proteolytic effect of ADAMTS5 was evident on C3 with molecular mass reduction as well as generation of additional fragments (fainter immunoreactive laddering; Fig. 5B). Importantly, fragmentation of C3 by ADAMTS5 caused clear changes in immunoreactivity and laddering when we probed with Abs against C3 neoepitopes C3C and C3D (Fig. 5B). The Ab against C3C detected fragmentation of C3 and laddering of a band reactive at the expected molecular mass of C3C (Fig. 5B; ∼137 kD). The Ab against C3D (Fig. 5B; ∼35 kD) detected modest laddering of immunoreactive bands and a new faint product below 25 kD. C4 and IgA appeared to be unaffected by ADAMTS5 (Fig. 5B).

We then sought to test whether ADAMTS5 alters the interaction of jacalin-purified IgA with mesangial cells. Primary, kidney mesangial cells were incubated with jacalin-purified IgA (from the serum of IgAN patients) with or without ADAMTS5 (Fig. 5C). Following treatment with IgA, there was observable accumulation of IgA and C3 on mesangial cell lysates (Fig. 5C) presumably due to interaction with cultured cells. ADAMTS5 reduced the immunoreactivity of IgA and C3 from mesangial cell lysates (Fig. 5C) and was able to generate ADAMTS-specific [14] DPEAAE VCAN fragments (Fig. 5C), indicating that the enzyme was proteolytically active in culture. Notably, the effect of ADAMTS5 was specific to the digestion of jacalin-purified IgA and its associated proteins rather than digestion of mesangial proteins by the enzyme (Fig. 5D). Pretreatment of mesangial cells for 18 h with ADAMTS5 (+TS5; lanes 2, 4, 6) or vehicle (veh; lanes 1, 3, 5) before adding jacalin-purified IgA (Fig. 5D; *+IgA* alone; lanes 3, 4), did not reduce the immunoreactivity of IgA or C3 detected in mesangial cell lysates. Instead, pretreatment of jacalin-purified IgA with ADAMTS5 for 18 h before adding on mesangial cells (Fig. 5D; *+IgA+TS5*; lanes 5, 6), reduced IgA and C3 immunoreactivity, in either vehicle or ADAMTS5-treated cells. Thus, ADAMTS5 enzymatic activity on jacalin-purified IgA and copurified proteins affected their interaction with mesangial cells. The protein or proteins (copurified with IgA using jacalin) that might be involved in binding of IgA immune complexes with mesangial cells are currently unknown, but it is likely that ADAMTS5 proteolysis will not be limited to FN1 and C3. Incubation of jacalin-purified IgA with ADAMTS5 did not affect activation of mesangial cells, at least in terms of IL-6 (Supplemental Fig. 3B) that was used in this study as a simple readout for the proinflammatory effect of IgA (37), indicating that neither ADAMTS5 nor fragmentation of jacalin-purified proteins by ADAMTS5 influenced IL-6 expression in vitro. Finally, incubation of jacalin-purified IgA with ADAMTS5 caused a similar reduction in IgA interaction on immortalized PTECs, further highlighting the reproducibility of this finding (Supplemental Fig. 3C, 3D). Thus, the effect of ADAMTS5 on one or more proteins copurified with IgA using jacalin, rather than processing of cellular proteins by the enzyme, appears to alter the interaction of IgA and associated proteins with cultured mesangial and proximal tubule cells. Excluding FN1 and C3 that were targeted in this study, other IgA associated proteins might also be affected.

We then used a commercially available complement pathway activity kit to examine whether in vitro digestion of C3 by ADAMTS5 alters its bioactivity. Incubation of jacalin-purified IgA with ADAMTS5 led to a significant reduction in classical pathway activation, whereas the lectin pathway was reduced but inconsistent (Fig. 5E). Alternative pathway activation was not detected, likely due to limitation in assay sensitivity. Given that these experiments rely on the digestion of IgA immune complexes with exogenous ADAMTS5 in vitro, more work is needed to understand their in vivo relevance and whether ADAMTS5 might alter the interaction of IgA immune complexes with kidney cells or the bioactivity of C3 in IgAN lesions. Presumably in vitro, ADAMTS5 cleaves complement proteins and this alters their overall bioactivity, but this might not be translated to the complex physiological conditions in vivo.

Finally, tissue staining revealed colocalization of C3 (blue) with ADAMTS5 (red) in IgAN biopsies in areas of inflammatory infiltration and matrix (VCAN; green) remodelling (Fig. 5F). This is perhaps expected given that C3 is expressed by macrophages (38), but highlights that the two proteins might coexist in inflammatory infiltrates. We also noted one kidney biopsy where ADAMTS5 colocalized with the anaphylatoxin C3A fragment (Fig. 5F; green) but this colocalization was unique to this specimen so this data should be treated with caution (more staining in Supplemental Fig. 4).

## Discussion

Collectively, we demonstrate the increase of ADAMTS5 in IgAN kidney biopsies in areas with inflammatory infiltration and matrix remodelling. ADAMTS5 is likely expressed in IgAN as well as other kidney pathologies, by infiltrating monocytes and might play a role in matrix turnover and scarring. The likely association of ADAMTS5 with infiltrating monocytes might explain the variable degree of immunostaining observed in different biopsies. Variable infiltration of peripheral monocytes in IgAN kidneys is not surprising and is related to differences in inflammatory and pathological processes as well as long-term outcome variability observed in IgAN patients (39, 40). Tubulointerstitial myeloid cell infiltration is associated with declining renal function in different nephropathies including IgAN (41–43). In support to this, ADAMTS5 staining was higher in patients with lower eGFR as well as in biopsies with more interstitial fibrosis (based on MEST-C score histological evaluation). Thus, it is likely that ADAMTS5 levels in IgAN biopsies reflect the variable tubulointerstitial inflammation and tissue pathology.

The expression of ADAMTS5 in kidney inflammation is unknown and perhaps more surprisingly, the expression of ADAMTS5 by immune cells in general has not been studied in depth. The function of ADAMTS5 in inflammation might receive increased attention, given that recent immune cell RNA-sequencing datasets show increased expression of the enzyme specifically in classical monocytes (25–27). Generally, proteolytic enzymes and metalloproteinases are commonly expressed and secreted by monocytes and other inflammatory cells (especially phagocytes) during pathological tissue remodelling in inflammatory and autoimmune diseases (44, 45) including IgAN (32, 33). The function of metalloproteinases is multimodal, but in tissue inflammation, they are classic effectors of monocyte infiltration by digesting the interstitial matrix (46). The possible role of ADAMTS5 in inflammatory infiltration in the kidney is supported in this study by the proteolytic effect that it exhibits on the renal matrix, in particular the basement membrane, which is important for normal kidney function. Digestion of basement-membrane proteins by activated monocytes is central in inflammatory cell infiltration and migration (47). Matrix proteolysis is also a typical feature of tissue inflammation and precedes fibrosis in different pathologies (48). Proteolysis of the basement membrane by metalloproteinases is also associated with fibrotic transformation of epithelial cells during tissue scarring (49, 50), including kidney tubulointerstitial inflammation and fibrosis (51).

The role of ADAMTS5 in C3 fragmentation needs to be investigated further in the context of IgAN inflammation and complement activation in vivo, given the importance of C3 in IgAN and inflammation in general. C3 proteolysis might generate active complement fragments (such as C3A) or render complement proteins inactive. For instance, although the possible function of ADAMTS5 on C3 is unknown, the ability of leukocyte elastase to process complement is known since the late 1970s (52), whereas a more recent study found that MMP12 was able to process and inactivate C3 in murine peritonitis (53). Although our data demonstrates that ADAMTS5 cleaves C3 and reduces complement activity in vitro, the effect of the enzyme might be different in vivo, under physiological or pathological conditions, where ADAMTS5 activity on different substrates (including C3) will be susceptible to multiple different cellular as well as soluble tissue and biofluid factors and natural proteinase inhibitors. For instance, α-2-macroglobulin, which is highly homologous to C3, is a substrate and inhibitor of ADAMTS5 (and other proteinases) and thus binding with such proteins might deactivate the enzyme.

ADAMTS5 might be a therapeutic target in kidney disease given that inflammatory kidney scarring is associated with loss of renal function. Pharmacologically, it might be more feasible to target a proteolytic enzyme rather than the entire inflammatory process, so targeting ADAMTS5 as a possible antiremodelling therapy could be relevant in IgAN and other kidney diseases with an inflammatory and scarring component. Indeed, the enzyme was not unique to IgAN as it was also expressed in membranous nephropathy and focal segmental glomerulosclerosis. The expression of ADAMTS5 in other kidney diseases is not surprising given that the enzyme is apparently enriched in classical monocytes. To this end, the potential renal and IgAN bioactivity of ADAMTS5 needs to be investigated in animal models focusing on kidney matrix remodelling and proteolysis, as enzymatic activity in vivo is likely to be different from the test-tube and cell culture experiments presented in this study. Although matrix proteolysis drugs are not yet mainstream, ADAMTS5 blocking is currently examined as a possible treatment in arthritis (54).

The possible inflammatory matrix remodelling role of ADAMTS5 in IgAN and kidney inflammation as well as its ability to fragment C3 is a promising area for future translational work. The unexpected enrichment of ADAMTS5 in monocytes together with evidence for its involvement in human organ disease warrant further investigation in diseases with inflammatory and fibrotic phenotypes.

## Supplementary Material

Data Supplement
