# Supplementary material for: The Metalloproteinase ADAMTS5 Is Expressed by Interstitial Inflammatory Cells in IgA Nephropathy and Is Proteolytically Active on the Kidney Matrix
Source: J Immunol. 2020 Sep 11;205(8):2243–54. doi: 10.4049/jimmunol.2000448 (PMC7533710; doi:10.4049/jimmunol.2000448)
Supplement: Data Supplement [file JI_2000448.zip › JI_2000448_Supplemental_Figures_1.pdf]

Supplemental Figure 1

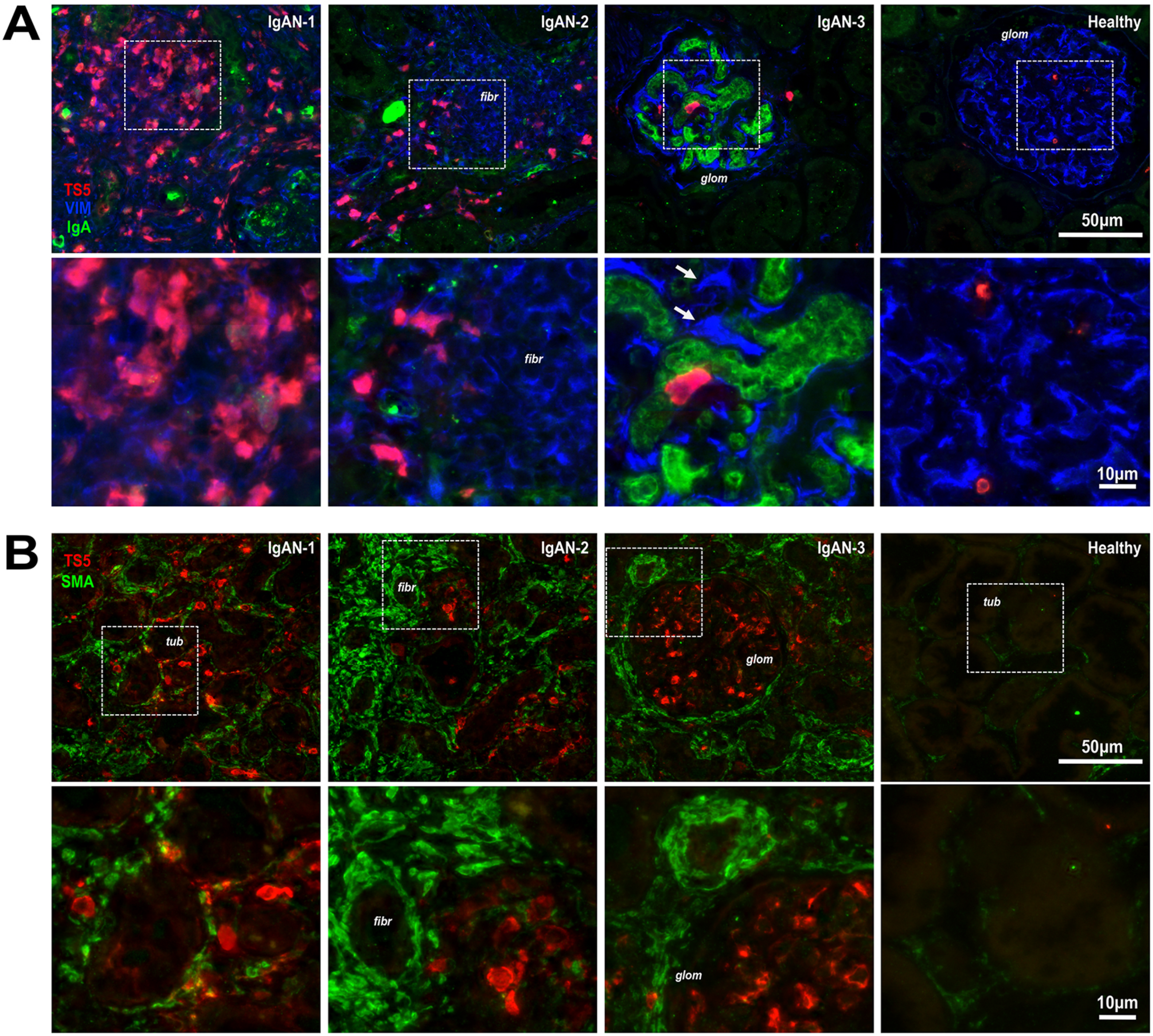

ADAMTS5 co-staining with Vimentin and Smooth Muscle Actin (SMA).

A: ADAMTS5 (red) was costained with the mesenchymal cell marker vimentin (blue) and IgA (green). ADAMTS5⁺ infiltrates express vimentin indicated by weak purple colour. Instead, ADAMTS5 is not expressed by vimentin⁺ cells (most likely mesangial) in the glomerulus (see IgAN-3; *glom*), nor by vimentin⁺ cells in fibrotic areas of the IgAN tubulointerstitium (see IgAN-2; *fibr*). ADAMTS5 does not colocalise with IgA deposits (green) around vimentin⁺ cells in glomeruli (see IgAN-3; *glom*).

B: ADAMTS5 (red) was costained with SMA, a marker of smooth muscle cells and myofibroblasts (green). ADAMTS5⁺ cells in the tubulointerstitium and glomeruli do not express SMA. Areas of fibrotic transformation that stain brightly with SMA (see green in IgAN-2 for example) have little ADAMTS5 expression.

White dashed boxes denote areas that are shown in higher magnification in lower panels.

Supplemental Figure 2

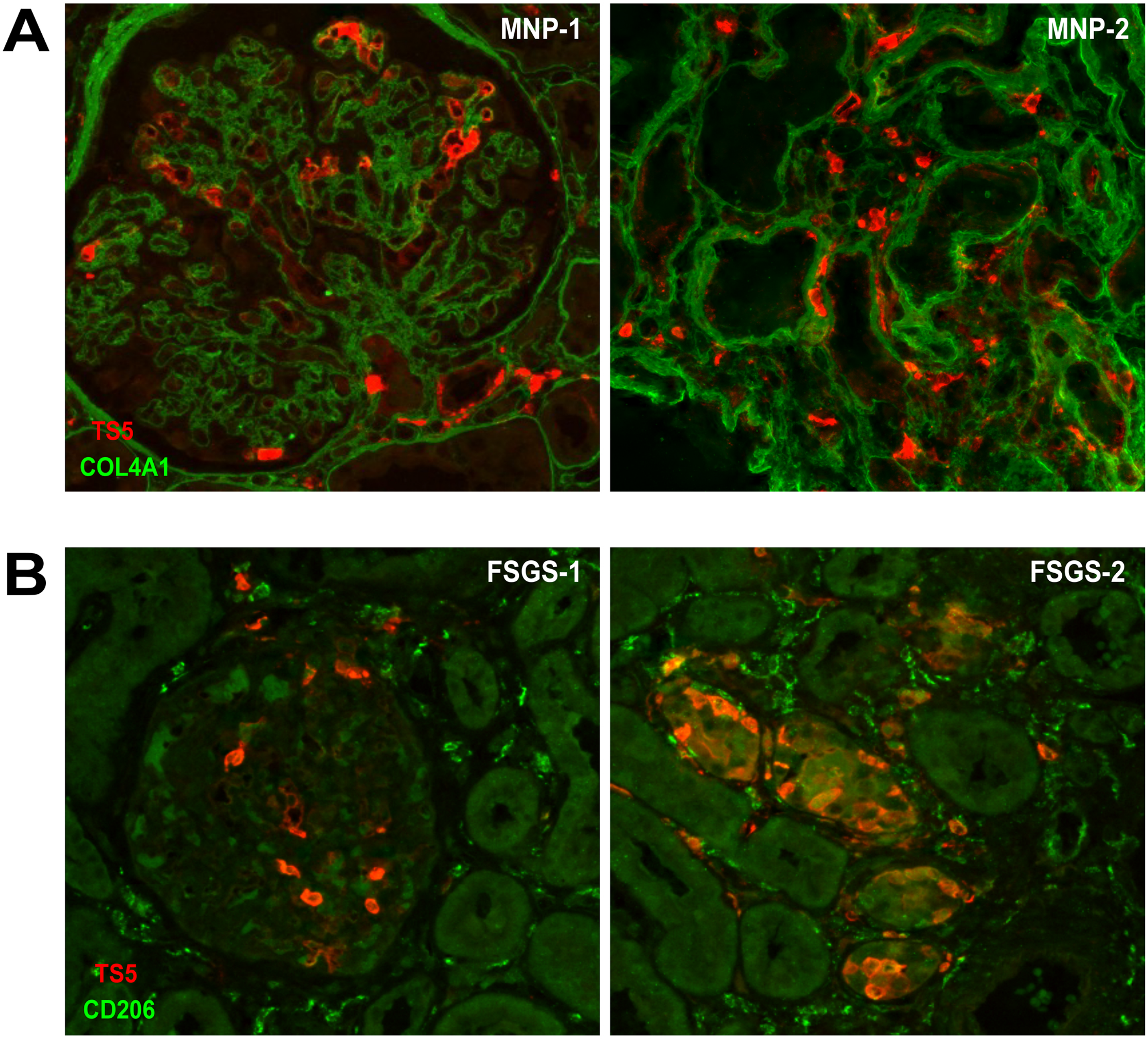

ADAMTS5 expression in other kidney diseases.

A-B: Tissue expression of ADAMTS5 was examined by immunostaining on membranous nephropathy (MNP; **A**) and focal segmental glomerulosclerosis biopsies (FSGS; **B**). In (**A**) ADAMTS5 was counterstained with COL4A1 (green) and in (**B**) with CD206 (green).

Supplemental Figure 3

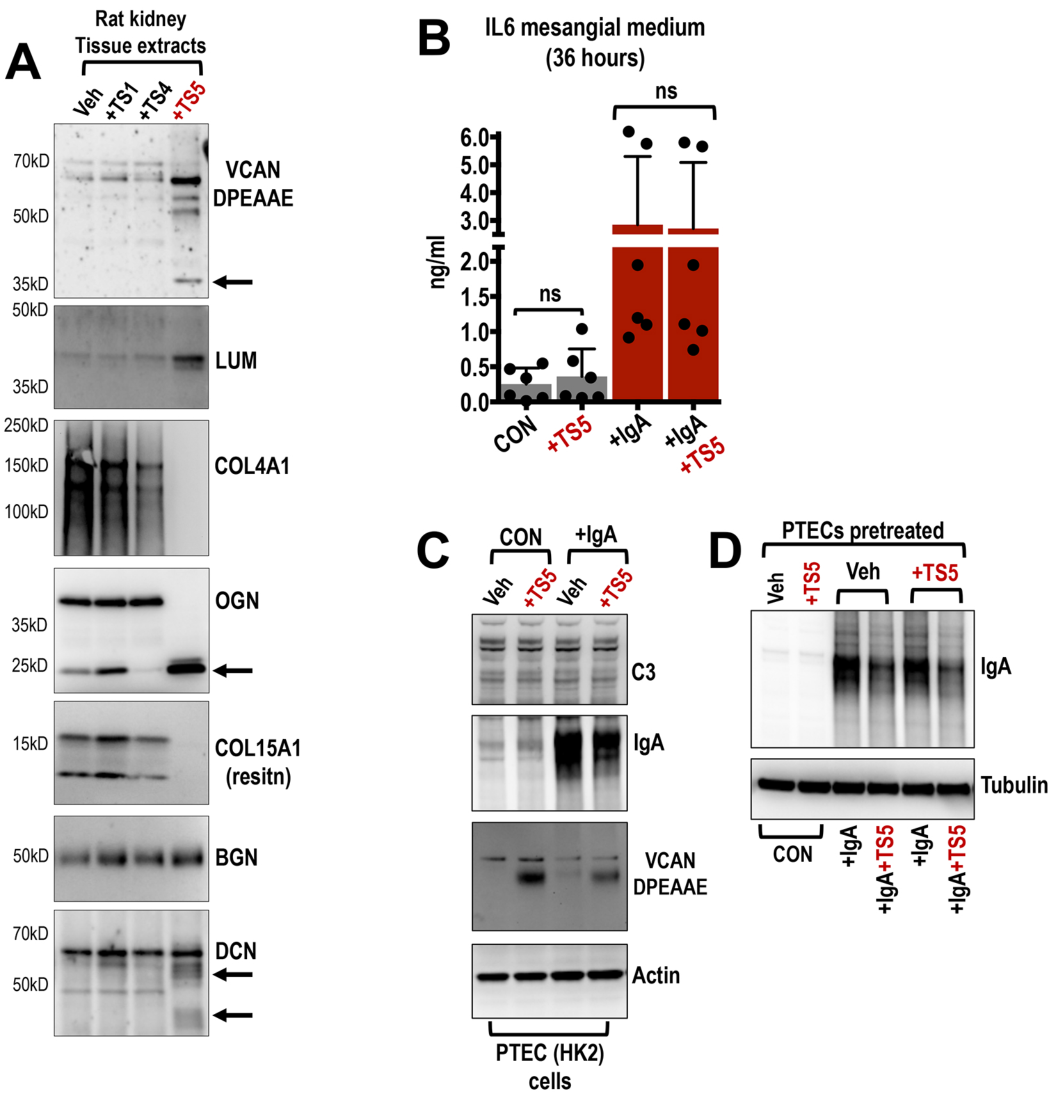

ADAMTS5 activity and cellular effects *in vitro*

A: Immunoblotting of proteins affected by enzymatic treatment of rat kidney explants using 4μg/ml of ADAMTS1, ADAMTS4 or ADAMTS5. Immunoblots were performed on 0.2% SDS protein extracts 36h after treatment of kidney explants. Control explants were incubated in enzyme digestion buffer only (CON). Collagen-4 (COL4A1) and collagen-15 (COL15A1) were completely processed by ADAMTS5. Osteoglycin (OGN) was degraded and replaced by a smaller fragment. Note that the antibody to versican (VCAN) is raised against the ADAMTS-generated DPEAAE versican neoepitope indicating robust generation of the fragment by ADAMTS5. Lumican (LUM), osteoglycin (OGN) and collagen-15 (COL15A1) were also detected in IgAN urine (see **Fig.1A**). For COL15 we only detected its small restin (or endostatin) fragments. Known ADAMTS substrates biglycan (BGN) and decorin (DCN) did not appear to be severely affected albeit DCN shows limited fragmentation.

B: IL6 ELISA in the culture medium of mesangial cells exposed to IgA with or without ADAMTS5 for 36h. Statistical analysis was performed using ANOVA with Fisher's LSD multiple comparison test. While there was a significant difference in IL6 levels ($p<0.01$) between controls and IgA-stimulated cells, treatment with ADAMTS5 had no effect on IL6 secretion in either control or IgA-stimulated cells.

C-D: Immunoblotting of immortalised proximal tubule epithelial cells (PTECs) for complement C3, IgA and versican DPEAAE neoepitopes **(B).** Cells were incubated with IgA purified from the serum of IgAN patients for 36 hours (+IgA), with or without ADAMTS5 (+TS5). Treatment of PTECs with jacalin-purified IgA causes accumulation of IgA on cell layers and treatment with ADAMTS5 causes a reduction. Unlike mesangial cells (see **Fig.5C-D** in main text) C3 does not appear to bind PTECs. The reduction in the binding of IgA on cultured cells is related to the digestion of jacalin-purified IgA by ADAMTS5 (+*IgA+TS5*) rather than pretreatment of cells by the enzyme **(C).**

Supplemental Figure 4

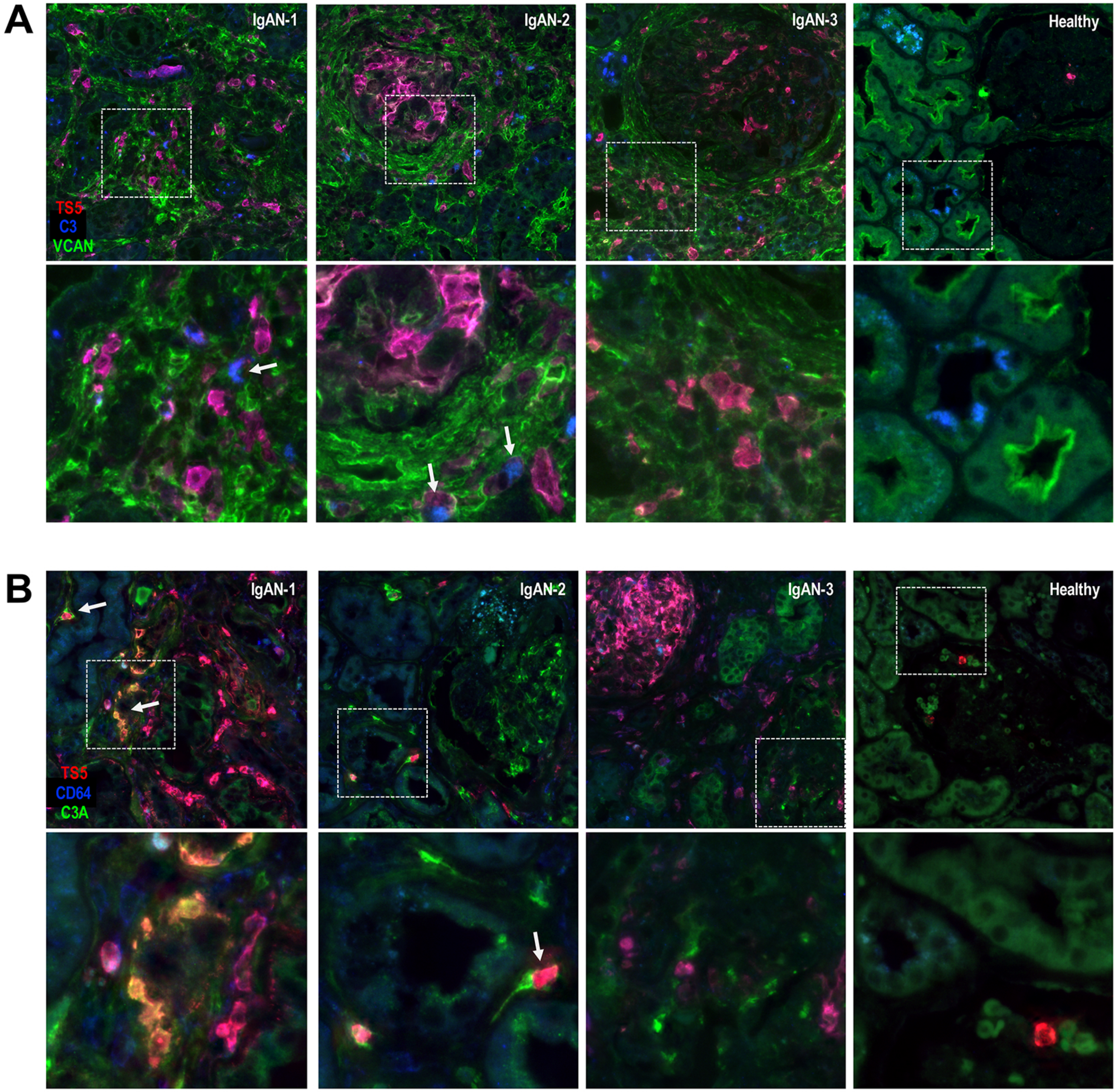

ADAMTS5 and Complement C3 tissue staining.

A: ADAMTS5 (red) was costained on IgAN and healthy biopsies with complement C3 (blue) and versican (VCAN; green). ADAMTS5 and C3 colocalise and positive cells are purple (mix of red and blue). There are also individual C3-stained structures without ADAMTS5 (see arrows).

B: ADAMTS5 (red) was costained on IgAN and healthy biopsies with CD64 (blue) and the C3 anaphylatoxin fragment C3A (green). We noticed an isolated spot of ADAMTS5 and C3A colocalisation in cells (see arrows) in one biopsy (IgAN-1).
